# Electrical Advantages of Dendritic Spines

**DOI:** 10.1371/journal.pone.0036007

**Published:** 2012-04-20

**Authors:** Allan T. Gulledge, Nicholas T. Carnevale, Greg J. Stuart

**Affiliations:** 1 Department of Physiology and Neurobiology, Geisel School of Medicine at Dartmouth, Hanover, New Hampshire, United States of America; 2 Department of Neurobiology, Yale University School of Medicine, New Haven, Connecticut, United States of America; 3 Eccles Institute of Neuroscience, John Curtin School of Medical Research, Australian National University, Canberra, Australian Capital Territory, Australia; Neuroscience Campus Amsterdam, VU University, Netherlands

## Abstract

Many neurons receive excitatory glutamatergic input almost exclusively onto dendritic spines. In the absence of spines, the amplitudes and kinetics of excitatory postsynaptic potentials (EPSPs) at the site of synaptic input are highly variable and depend on dendritic location. We hypothesized that dendritic spines standardize the local geometry at the site of synaptic input, thereby reducing location-dependent variability of local EPSP properties. We tested this hypothesis using computational models of simplified and morphologically realistic spiny neurons that allow direct comparison of EPSPs generated on spine heads with EPSPs generated on dendritic shafts at the same dendritic locations. In all morphologies tested, spines greatly reduced location-dependent variability of local EPSP amplitude and kinetics, while having minimal impact on EPSPs measured at the soma. Spine-dependent standardization of local EPSP properties persisted across a range of physiologically relevant spine neck resistances, and in models with variable neck resistances. By reducing the variability of local EPSPs, spines standardized synaptic activation of NMDA receptors and voltage-gated calcium channels. Furthermore, spines enhanced activation of NMDA receptors and facilitated the generation of NMDA spikes and axonal action potentials in response to synaptic input. Finally, we show that dynamic regulation of spine neck geometry can preserve local EPSP properties following plasticity-driven changes in synaptic strength, but is inefficient in modifying the amplitude of EPSPs in other cellular compartments. These observations suggest that one function of dendritic spines is to standardize local EPSP properties throughout the dendritic tree, thereby allowing neurons to use similar voltage-sensitive postsynaptic mechanisms at all dendritic locations.

## Introduction

Spines are prominent postsynaptic morphological features found on the dendrites of many neurons. Many functions for spines have been proposed, including electrical filtering and isolation of synaptic inputs [1]–[5], chemical compartmentalization [6]–[10], and maximization of the number of potential synaptic connections [11], [12]. However, despite more than a century of research, a definitive role for dendritic spines remains elusive.

Excitatory postsynaptic potentials (EPSPs) are shaped locally by the dendritic geometry at the site of synaptic input [13], [14]. EPSPs tend to have larger amplitudes and faster kinetics when generated in neuronal compartments with higher input impedance and smaller local capacitance, as typically occurs at distal locations within dendritic trees. As a result, local EPSPs at the site of synaptic input can be highly variable in their amplitude and kinetics [15].

**Figure 1:**
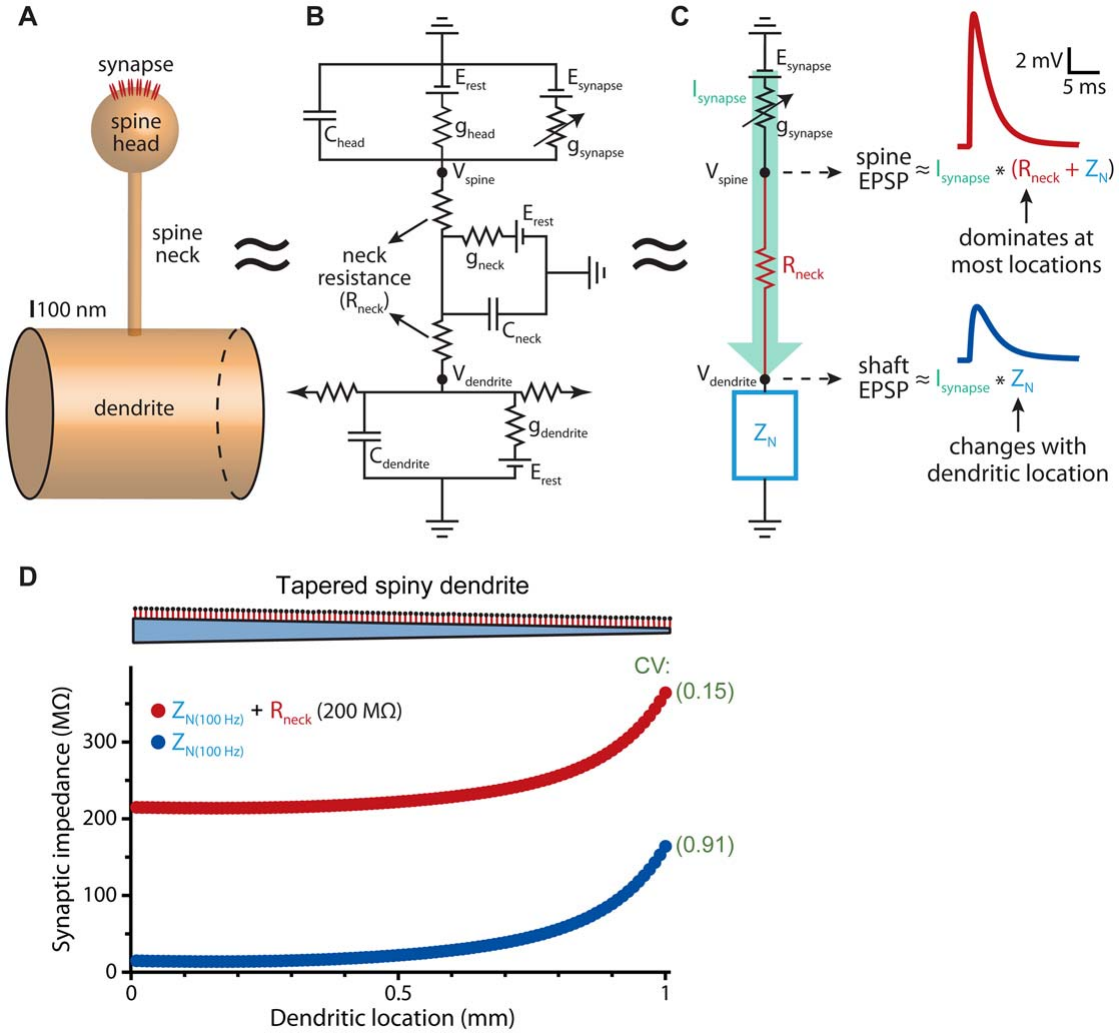
Electrical properties of dendritic spines. A) Diagram of a dendritic spine consisting of a spine “head" attached to a dendrite by a narrower spine “neck". B) Spines can be modeled as a series of electrical compartments, each having membrane conductance (g_head_, g_neck_, and g_dendrite_) and capacitance (C_head_, C_neck_, and C_dendrite_) determined by the surface area of the compartment. Internal “axial" resistance between compartments reflects the conductivity of the cytoplasm and the morphology (cross-sectional area and length) of the communicating compartments. The small surface area of spines minimizes their membrane resistance and conductance, allowing simplification of the electrical structure of spines (C), in which synaptic current (I_synapse_) is illustrated in green, and where dendritic electrical properties, including dendritic connectivity with the rest of the neuron, are represented in aggregate as “input impedance" (Z_N_; blue), a measure analogous to input resistance, but also incorporating capacitive influences on non-steady-state voltage signals such as synaptic potentials. EPSPs in spines approximate the product of I_synapse_ and the “in series" sum of R_neck_ and Z_N_ (R_head_ being a negligible “in parallel" resistance to synaptic current). On the other hand, shaft EPSPs, whether generated by synaptic current originating in spine heads or from synapses located on the dendritic shaft, will vary with the product of I_synapse_ and Z_N_. D) Plot of Z_N_ (calculated for 100 Hz input) and Z_N_+R_neck_ (for 200 MΩ spine necks) verses distance along a tapering (5 µm to 1 µm) 1000 µm-long dendrite (cartoon at top not to scale) attached to a 40 µm by 40 µm soma (not shown). Spines with neck resistances of 200 MΩ were placed every 10 µm along the dendrite. Coefficients of variation (CV) for Z_N_ and Z_N_+R_neck_ values indicated in green. EPSPs shown in part C are from the simulations depicted in Figure 2A for a spine input at the distal end of the dendrite.

In spiny neurons, excitatory synapses occur on dendritic spines. Spines have distinct electrical properties that shape synaptic responses locally at the site of synaptic input, but have little impact on EPSPs recorded in dendrites or at the soma [16]–[19]. Spines consist of a spine “head", onto which excitatory synapses are made, and a spine “neck" that attaches the spine head to the dendritic shaft (Figure 1A). Each of these “compartments" can be modeled as electrical circuits (Figure 1B) having conductance and capacitance determined by the surface area of the surrounding plasma membrane. The small surface area of spines (<1 µm^2^) provides negligible local membrane conductance and capacitance, and as such almost all the synaptic current entering a spine is transferred to the dendritic shaft via the spine neck resistance (Figure 1C) [16]. Because EPSPs are the product of synaptic current and resistance to that current (Ohm's law), the amplitude of synaptic responses in the spine head will depend in large part on the “in series" sum of spine neck resistance and dendritic input impedance (Z_N_; see Figure 1 legend). Z_N_ varies with dendritic geometry and topography, and at most dendritic locations is expected to be much lower than spine neck resistance (Figure 1D). This could limit the influence of dendritic location on spine EPSP amplitudes. On the other hand, EPSPs in dendrites have amplitudes determined by the product of the synaptic current and Z_N_ alone, which should generate EPSPs that are smaller and more location-dependent than those occurring in synaptically activated spine heads. Finally, since spines have little impact on the synaptic current entering dendrites [16], dendritic EPSPs generated by spine synapses will appear similar to those generated by synapses located directly on the dendritic shaft.

The inability of spines to significantly shape EPSPs recorded in dendrites or at the soma has led some authors to question whether spines provide electrical advantages to neurons [20]. Yet, from the point of view of the synaptic membrane, where numerous voltage-sensitive mechanisms may exist [21]–[24], spine necks play a critical role in shaping EPSPs. In this paper we use computational models of simplified and morphologically realistic dendritic trees to directly compare synaptic responses in spines and dendritic shafts to test the hypothesis that spines act to limit location-dependent variability of EPSP properties at the site of synaptic input. Such comparisons in real neurons are not possible given technological limitations of electrical recording and imaging techniques [25], and the rarity of excitatory inputs onto dendritic shafts in spiny neurons [26]. By simulating identical synaptic inputs onto spines and shafts at all dendritic locations, we demonstrate that spine morphologies standardize the amplitude and kinetics of local EPSPs, limiting their dependence on synapse location within the dendritic tree, and allowing more uniform activation of voltage-sensitive conductances at the site of synaptic input. Because spines reduce the impact of local dendritic geometry on EPSP properties, they may allow neurons to use similar voltage-sensitive postsynaptic mechanisms at all excitatory synapses, regardless of their location in the dendritic tree.

## Results

### Spines standardize EPSP properties in a simplified model neuron

**Figure 2:**
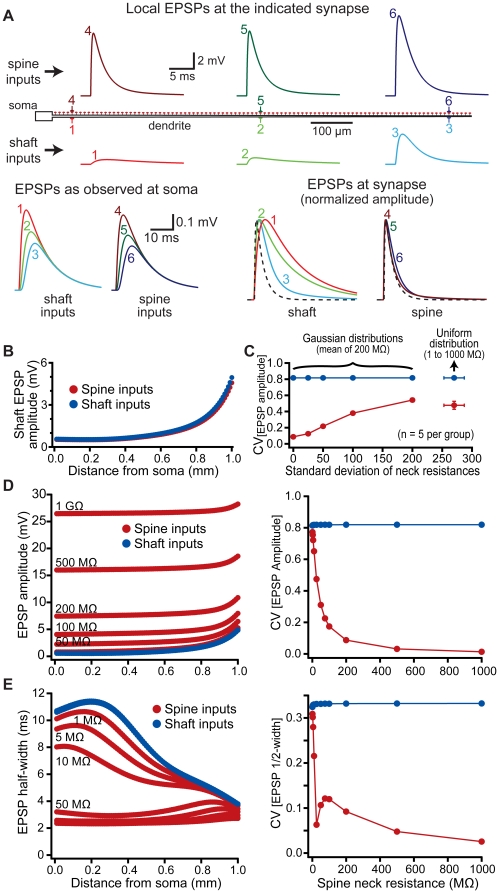
Spines reduce location-dependent variability of EPSP properties. A) Top, diagram of a “ball-and-stick" model neuron. Synapses were placed onto the dendritic shaft or onto spines (200 MΩ spine neck resistance) at the locations indicated (synapses 1 to 6). Local EPSPs generated in the dendritic shaft (synapses 1 to 3, lower traces) or in spines (synapses 4 to 6, upper traces) are color-coded by location. Bottom left, somatic EPSPs resulting from inputs to the shaft (1 to 3) and spines (4 to 6). Bottom right, local shaft (1 to 3) and spine (4 to 6) EPSPs normalized and superimposed to allow comparison of EPSP kinetics. The time course of the underlying synaptic conductance is indicated by a dashed line. B) Plots of EPSP amplitudes for spine (red) and shaft (blue) inputs as measured in the dendritic shaft. C) Plot of the coefficients of variation (CVs) (mean ± standard deviation) for spine (red) and shaft (blue) EPSP amplitudes for inputs having variable spine neck resistances determined from Gaussian or uniform distributions, as indicated (n = 5 trials per group). D, E) Left, plots of local EPSP amplitude (D) and EPSP half-width (E) versus distance from the soma for inputs onto the dendritic shaft (blue) and spines (red) with the indicated spine neck resistances. Right, plots of the coefficient of variation (CV) for EPSP amplitude (D) and half-width (E) versus spine neck resistance.

We initially tested the electrical consequences of spines in a simplified “ball-and-stick" model (see Methods) in which AMPA-like synaptic conductances were generated on spines (200 MΩ neck resistance) or onto the neighboring dendritic shaft at evenly spaced dendritic locations (Figure 2). Voltage responses were recorded locally at the site of synaptic input (i.e., in the spine head for spinous inputs or in the adjacent dendritic shaft for dendritic inputs), at the soma, and, in the case of spine inputs, in the shaft below the spine. As expected [15], the amplitude and kinetics of local EPSPs occurring on dendritic shafts were highly location-dependent, tending to be larger and faster at locations distal from the soma (Figure 2A). In contrast, local EPSPs occurring on dendritic spines were more uniform in their amplitude and kinetics across dendritic locations (Figure 2A). This effect of spines on EPSPs was restricted to the site of synaptic input, as spine EPSPs measured within the dendritic shaft or at the soma were similar, although slightly smaller, than shaft EPSPs generated at the same dendritic locations (Figure 2A and B). Spine-dependent standardization of EPSP properties at the site of synaptic input persisted even when the resistance of individual spine necks was varied around a mean value using Gaussian or uniform distributions (Figure 2C).

Variability in EPSP properties was quantified by calculating the coefficient of variation (CV) of local EPSP amplitudes and half-widths for inputs on shafts or onto spines having a range of spine neck resistances (Figure 2D and E). EPSPs on spines were less variable than were EPSPs generated on dendritic shafts at the same dendritic locations over a range of spine neck resistances. The influence of spines on local EPSP properties was largely independent of synaptic conductance (range examined: 250 pS to 2 nS) or spine head diameter, but depended heavily on spine neck resistance (range examined: 1 to 1000 MΩ). Higher spine neck resistances generated larger and faster local EPSPs in spines at all dendritic locations, leading to reduced location-dependent variability of local EPSP properties (Figure 2D and E). Yet, even with relatively low spine neck resistances (as low as 10 MΩ), the CVs of spine EPSP amplitude (0.65) and half-width (0.22) were lower for inputs onto spines when compared to those onto dendritic shafts at the same dendritic locations (0.82 and 0.33, respectively).

**Figure 3:**
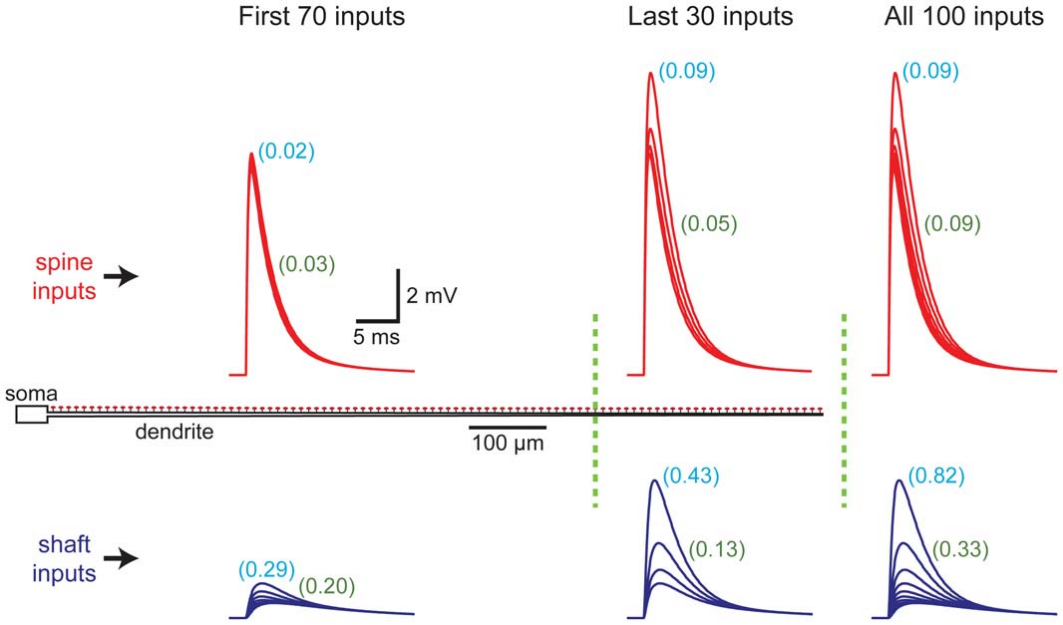
Spine-dependent reduction of EPSP variability does not depend on dendritic location. Comparisons of the CVs for EPSP properties calculated over the entire dendritic population, or for inputs restricted to the first 700 µm (70% of inputs) or last 300 µm (30% of inputs) of the dendrite. Left, local EPSPs at spine (red traces) or shaft (blue traces) inputs along the first 700 µm (∼100 µm intervals). Middle, local spine and shaft EPSPs generated along the last 300 µm of dendrite. Right, local EPSPs along the entire dendrite. CVs for EPSP amplitude (indicted in light blue) and half-width (indicated in green) were calculated for all inputs located within the dendritic subregions (n = 70, 30, and 100, respectively, for first 70%, last 30%, and entire synapse population).

Spine-dependent standardization of EPSP properties at the site of synaptic input was not dependent on the increased variability evident at distal dendritic locations (Figure 3). When considering inputs onto the entire dendrite, the CVs of EPSP amplitude and half-width equaled 0.09 for spine inputs (spine neck resistance = 200 MΩ), but were approximately 9 fold (CV_[EPSP amplitude]_ = 0.82) and 3 fold (CV_[EPSP half-width]_ = 0.33) higher for shaft inputs. When considering only those inputs occurring along the first 70% (700 µm) of the dendrite, CVs for EPSP amplitude and half-width were 0.02 and 0.03, respectively, for spine inputs, and 0.29 and 0.20 for shaft inputs; differences of almost 15 and 7 fold, respectively. On the other hand, for the most distal 30% (300 µm) of the dendrite, CVs calculated for EPSP amplitudes and half-widths were 0.09 and 0.05, respectively, for spine inputs, and 0.43 and 0.13, respectively, for shaft inputs; differences of almost 5 and 3 fold, respectively. We conclude that spine-dependent reductions in the variability of EPSP properties occurs over the entire dendrite, and does not depend upon “end effects" occurring at the tips of dendrites.

### Spines standardize EPSP properties in morphologically realistic models

**Figure 4:**
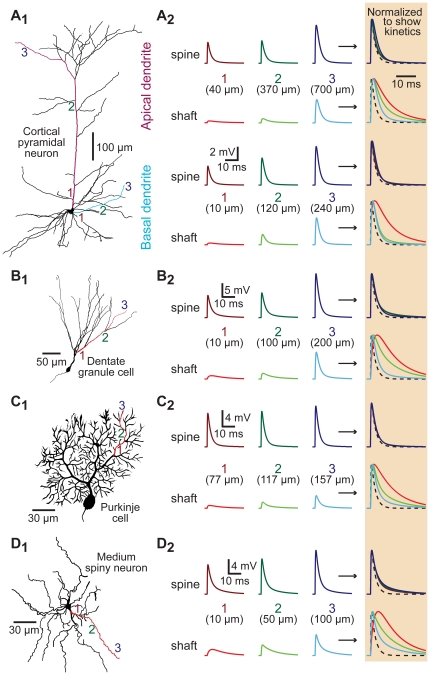
Spines standardize EPSP properties in morphologically realistic neurons. A_1_–D_1_) Morphology of reconstructed neurons: A_1_) layer 5 pyramidal neuron from the medial prefrontal cortex, B_1_) hippocampal dentate granule cell, C_1_) cerebellar Purkinje neuron, and D_1_) striatal medium spiny neuron. Synaptic inputs were placed onto shafts and spines of the colored dendrites at proximal, intermediate, and distal locations as indicated by the numbered locations (1 to 3). A_2_–D_2_) Left, local EPSPs recorded in spines (top traces) or in dendritic shafts (lower traces) at the locations indicated in the different morphologies. Normalized and superimposed traces, expanded in time and shaded at far right, allow comparison of EPSP kinetics. The time course of the underlying synaptic conductance is indicated by dashed lines.

To test whether spines standardize EPSP properties in morphologically realistic neurons, we utilized 3-dimensional reconstructions of several types of spiny neurons (Figure 4). In each model, spines (200 MΩ neck resistance) were placed at ∼10 µm intervals along all spiny dendrites and EPSPs generated either in spine heads or on dendritic shafts adjacent to spines. As was found in the ball-and-stick model, spines decreased the location-dependent variability of local EPSP amplitude and kinetics in the apical and basal dendrites of a layer 5 pyramidal neuron from the prefrontal cortex (Figure 4A), as well as in the dendrites of a hippocampal dentate granule cell (Figure 4B), a cerebellar Purkinje neuron (Figure 4C), and a striatal medium-spiny neuron (Figure 4D). The CVs of EPSP properties were measured across all shaft and spine synapses for each of the different dendritic morphologies. This analysis indicated that spines significantly (p<0.001; repeated measures ANOVA) reduced distance-dependent variability in local EPSP amplitude and half-width, confirming that spines act to standardize EPSP amplitudes and kinetics at the site of synaptic input in morphologically realistic dendritic trees.

### Spines standardize activation of voltage-gated calcium channels

**Figure 5:**
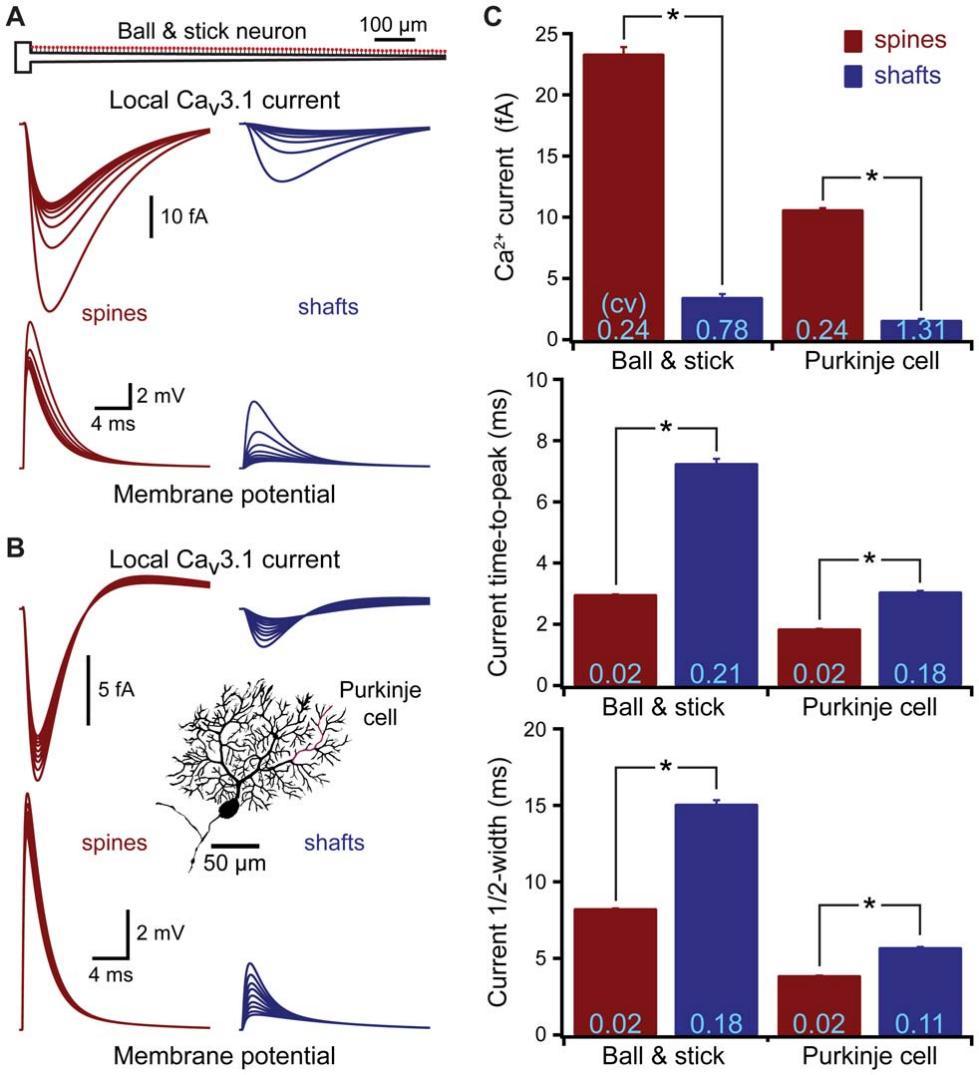
Spines standardize synaptic activation of voltage-gated calcium channels. A) Top, ball and stick model neuron. Local EPSPs (bottom) and calcium currents (middle) generated by inputs onto spines (left) or dendritic shafts (right) located at ∼100 µm intervals along the dendrite, each synapse contains the equivalent of ten Ca_v_3.1 (T-type) calcium channels (total maximum conductance, 50 pS). B) Local EPSPs (bottom) and calcium currents (top) generated by inputs onto spines (left) and shafts (right) located at ∼10 µm intervals along a spiny dendrite (red) of a cerebellar Purkinje neuron (inset). C) Average calcium current amplitude, time-to-peak, and half-width for all spine (red) and shaft (blue) inputs in the ball and stick (n = 100 inputs) and Purkinje neuron (n = 367 inputs) models. Asterisks indicate p<0.0001 (paired t-tests). CVs are indicated in light blue at base of each bar.

Synaptic transmission can involve postsynaptic voltage-sensitive processes that may benefit from spine-dependent standardization of EPSP amplitude and kinetics. One mechanism present at many synapses are low-threshold (i.e.,“T-type") voltage-gated calcium-channels (VGCCs) that provide a source of postsynaptic calcium and depolarization. We first tested the ability of EPSPs to activate T-type VGCCs at synapses occurring on spines or onto the dendritic shaft in a ball-and-stick model (Figure 5A). The equivalent of ten Ca_v_3.1 (T-type) channels (50 pS maximum combined conductance) was placed at spine and shaft synapses localized at ∼10 µm intervals along the dendrite. Synapses on spines generated larger and less variable postsynaptic calcium currents than did synapses occurring on the dendritic shaft, with current amplitude, time-to-peak, and half-width all having lower variation when inputs occurred on spines (Figure 5C). Since Ca_v_3.1 channels are known to be localized to spines in cerebellar Purkinje neurons [21], we tested synaptic T-type channel activation in a Purkinje neuron model having shaft and spine inputs placed at ∼10 µm intervals from the soma (Figure 5B). As was found in the ball-and-stick model, EPSPs occurring in spines generated larger and less variable calcium currents than did EPSPs on dendritic shafts (Figure 5B and C). Similar results were observed in models of a medium-spiny neuron, a dentate granule cell, and a layer 5 pyramidal neuron (not shown).

### Spines facilitate and reduce the variability of AMPA-dependent NMDA currents

**Figure 6:**
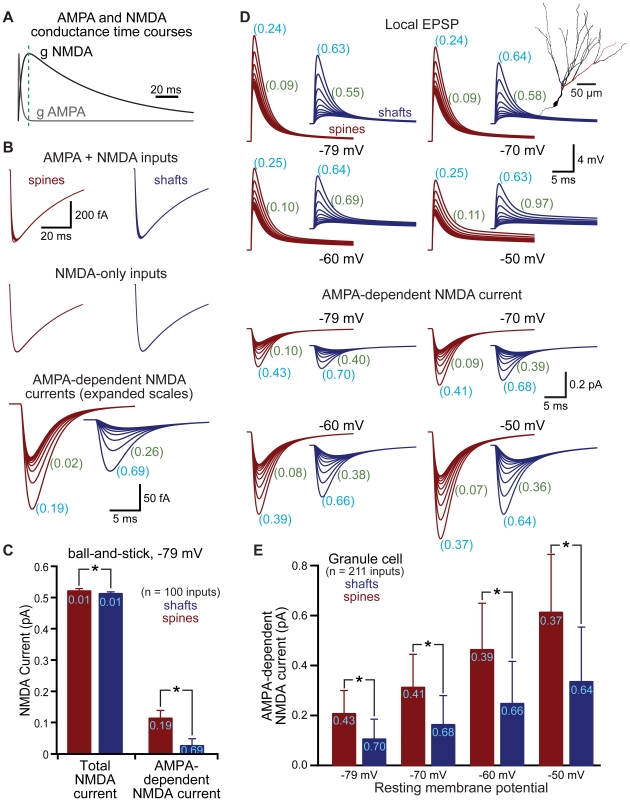
Spines enhance and standardize AMPA-dependent NMDA currents. A) Time courses of AMPA and NMDA receptor-mediated conductances. Green dashed line indicates the peak of the slower NMDA conductance. B) NMDA currents generated in a ball-and-stick model neuron when both AMPA and NMDA conductances are activated (top) or when the NMDA conductance is activated alone (middle). Subtraction allows isolation of the AMPA-dependent NMDA current (bottom). Traces show responses at ∼100 µm intervals. C) Comparison of total NMDA current (left) and AMPA-dependent NMDA current (right) in spines (red) and shafts (blue) for the ball-and-stick neuron resting at −79 mV. CVs shown in light blue. D) Local (spine or shaft) EPSPs (top traces) and AMPA-dependent NMDA currents (lower traces) simulated in a dentate granule neuron (inset). Traces show responses at inputs occurring at ∼20 µm intervals along the dendrite indicated in red in the inset morphology. CVs for EPSP or AMPA-dependent NMDA current amplitudes (light blue) or half-widths (green) shown for all 211 inputs (∼10 µm intervals) throughout the granule cell dendritic tree. E) Comparison of AMPA-dependent NMDA current amplitudes for inputs onto spines (red) and shafts (blue) in a dentate granule cell at the indicated resting membrane potentials. CVs shown in light blue. Data shown as mean ± standard deviation. Asterisks indicate p<0.05.

Another important postsynaptic mechanism present in many cell types is the NMDA-type glutamate receptor, which is voltage sensitive due to block by magnesium at hyperpolarized membrane potentials. By standardizing local AMPA EPSP amplitude and kinetics, spines might be expected to generate more uniform NMDA-mediated responses within dendritic trees. However, the kinetics of NMDA receptors are much slower than those of AMPA receptors (Figure 6A; see also [27]), limiting the potential influence of fast AMPA-mediated responses on NMDA currents. To test the impact of spines on NMDA receptor currents, we simulated NMDA-like conductances in spines and shafts, either alone, or together with an AMPA-like conductance, in a ball-and-stick model (Figure 6B and C) and in a model of a dentate granule cell (Figure 6D and E). Placing inputs onto spines led to small but significant increases in total NMDA currents, and standardized the amplitude and half-width of the AMPA-dependent component of NMDA currents (Figure 6B–E). In both the simplified ball-and-stick model (not shown) and the dentate granule cell (Figure 6D and E), we measured AMPA-dependent NMDA currents at several resting membrane potentials (−79, −70, −60, and −50 mV). AMPA-dependent NMDA currents in spines were larger and more uniform than were those generated at shaft synapses over this range of membrane potentials. These simulations indicate that spines boost and standardize local AMPA-driven activation of NMDA conductances during synaptic transmission.

### Spines promote initiation of NMDA-spikes

Voltage-sensitive conductances endow neurons with non-linear properties that enhance their computational capacity. NMDA receptors provide an important mechanism for non-linear synaptic integration in pyramidal neurons [28]–[31], where coactivation of a sufficient number of excitatory inputs on a dendritic branch can generate an “NMDA-spike", resulting in supra-linear summation of excitatory input at the soma.

**Figure 7:**
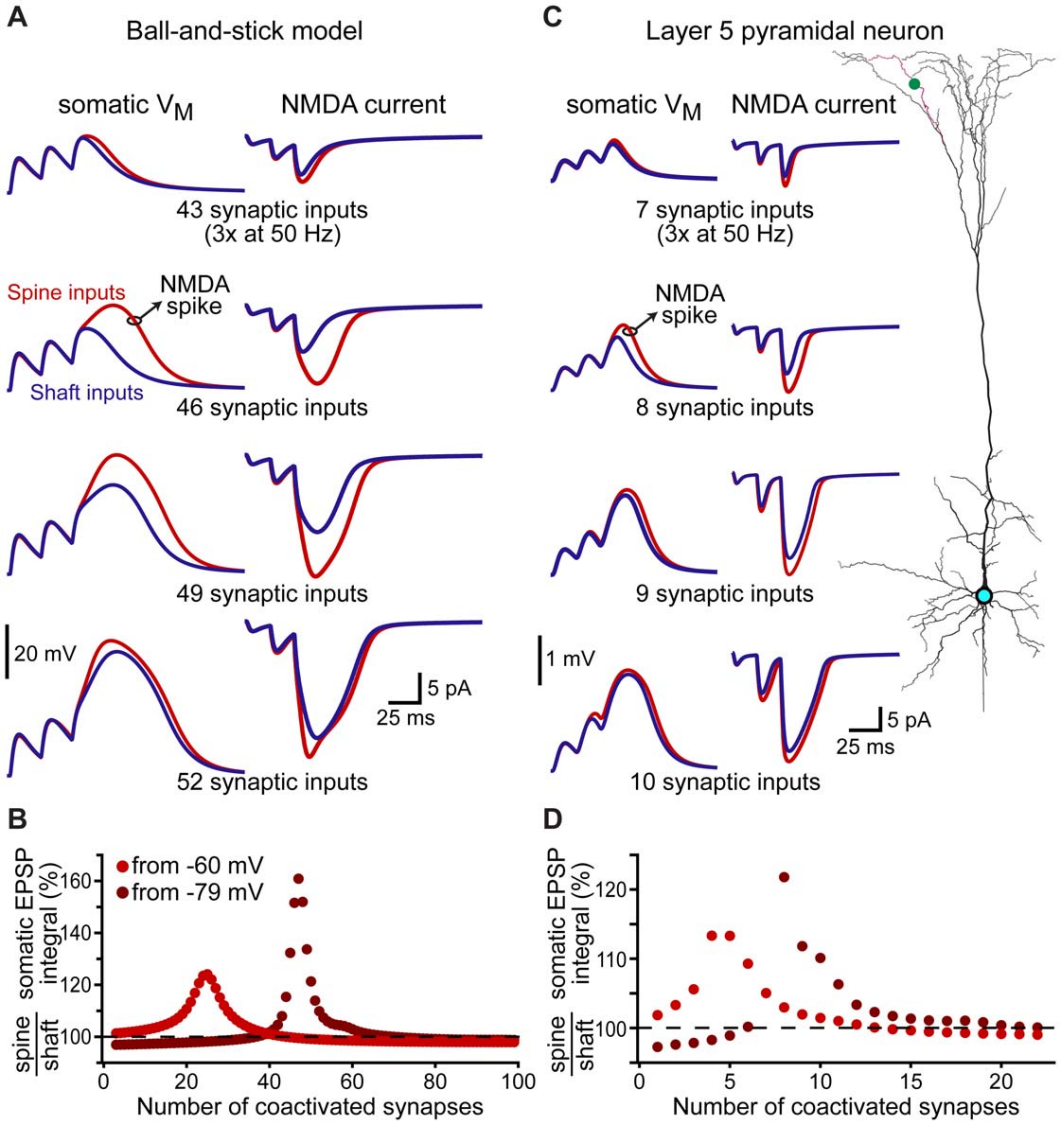
Spines lower the threshold for NMDA spike generation. A) Somatic voltage (left) and NMDA current (right) evoked by simultaneous activation of different numbers of AMPA+NMDA inputs (trains of 3 activations at 50 Hz) at synaptic locations evenly distributed along the dendrite of a ball-and-stick model resting at −79 mV. For each trial, NMDA currents were recorded from the synaptic input closest to the half-way point along the dendrite. Blue traces reflect responses to shaft inputs, while red traces are responses to spine inputs. B) Plot of the ratios of somatic EPSP integrals (spine inputs/shaft inputs) for trains of different numbers of evenly distributed inputs when the resting potential was set to −79 mV (brown) or −60 mV (red). C) Somatic voltage (left) and NMDA currents (right) evoked by trains of different numbers of AMPA+NMDA synaptic inputs (3 activations at 50 Hz) evenly distributed along the indicated apical branch of a layer 5 pyramidal neuron (right; red dendrite, green dot placed at half-way point along branch). D) Summary plot of the ratios of somatic EPSP integrals (spine inputs/shaft inputs) for trains of different numbers of coactivated inputs in the layer 5 pyramidal neuron resting at −79 mV (brown) or −60 mV (red).

**Figure 8:**
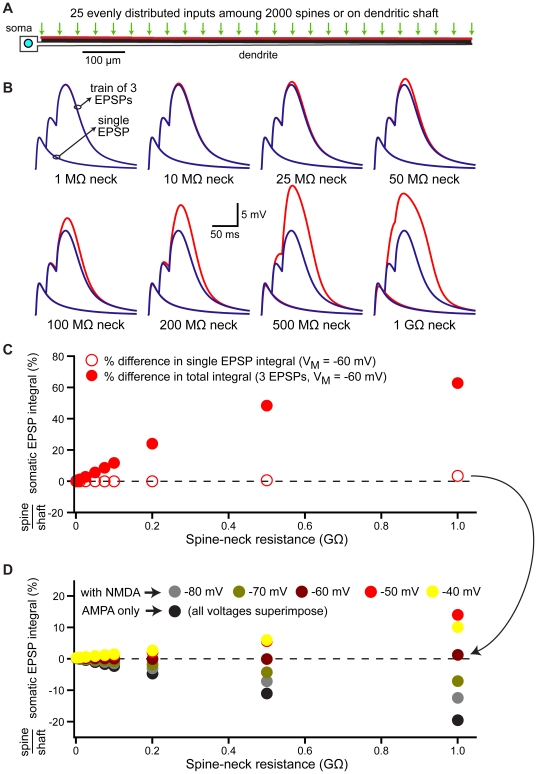
Spines enhance NMDA receptor activation over a variety of spine-neck resistances. A) Diagram of a ball-and-stick model neuron with 25 coactivated AMPA+NMDA inputs dispersed evenly among 2000 dendritic spines or directly over the dendritic shaft. Resting V_M_ is −60 mV. B) Somatic responses to single EPSPs or trains of three EPSPs generated at spine (red) or shaft (blue) inputs for models with the indicated spine neck resistances. C) Ratios of somatic EPSP integrals (spine inputs/shaft inputs) for single (open circles) and trains of three (filled circles) EPSPs generated by 25 distributed inputs in models with different spine neck resistances. D) Ratios of somatic EPSP integrals (spine inputs/shaft inputs) for single EPSPs generated by 25 distributed inputs in models with different spine neck resistances and resting membrane potentials. Resting V_M_ as indicated in color chart.

Because synapses on spines generate larger NMDA currents than do inputs onto shafts (see Figure 6), we tested whether spines promote the initiation of NMDA spikes in a generic ball-and-stick model (Figure 7A and B), and in the apical tuft of a layer 5 pyramidal neuron (Figure 7C and D). In both models, increasing numbers of synaptic inputs distributed evenly along the tested dendrite were activated together three times at 50 Hz; a protocol previously shown to reliably generate NMDA spikes in pyramidal neuron dendrites [30]. EPSPs in response to activation of spine or shaft inputs were recorded at the soma (Figure 7A and C), while NMDA currents were recorded at a synapse located approximately half-way along the dendritic branch (Figure 7A and C). When synaptic inputs were localized on spines, fewer coactivated inputs were required to initiate NMDA-dependent supra-linear depolarization of the soma. Spine inputs generated larger somatic depolarizations over a broad range of synaptic input (Figure 7B and D), even when resting potentials were set to more depolarized levels (−60 mV rather than −79 mV). Spine-dependent amplification of somatic depolarization occurred over a range of physiologically relevant spine neck resistances (Figure 8), suggesting that non-linear amplification of synaptic responses may be a key function of dendritic spines.

### Active dendritic conductances enhance spine-dependent standardization of EPSPs

**Figure 9:**
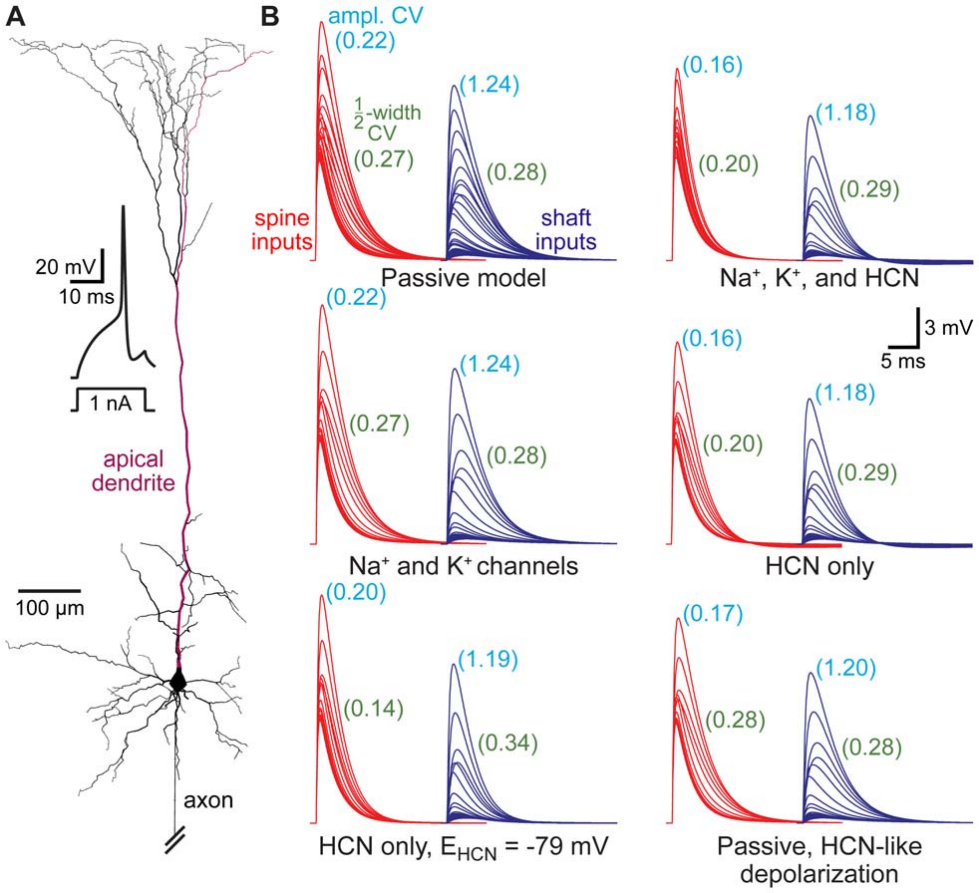
Dendritic HCN channels enhance spine-dependent standardization of EPSPs. A) Reconstructed layer 5 pyramidal neuron from the somatosensory cortex with spines at ∼10 µm intervals throughout the dendritic tree. Inset, action potential generated in an “active model" containing sodium, potassium, and HCN channels. B) EPSPs generated in spines (red) or shafts (blue) at ∼50 µm intervals along the apical dendrite (red dendrite in A) in models with different passive and active properties. Numbers in light blue and green indicate coefficients of variation (CVs) for EPSP amplitudes and half-widths, respectively, for all local responses (10 µm intervals) to spine and shaft inputs in the various models.

The dendrites of many neurons express voltage-gated ion channels that dynamically regulate neuronal excitability and synaptic integration. We investigated the impact of active dendritic conductances on spine-dependent standardization of EPSP properties in a model of a somatosensory layer 5 pyramidal neuron (Figure 9A) having well characterized dendritic properties [32]. Synaptic inputs activating AMPA and NMDA receptors were placed onto spines or on the dendritic shaft at ∼10 µm intervals throughout the dendritic tree, and inputs along an apical dendrite were individually activated. In the absence of dendritic voltage-gated ion channels, CVs for EPSP amplitude and half-width were lower for spinous inputs than for inputs made at the same locations on the dendritic shaft (Figure 9B; “Passive model"). The addition of dendritic voltage-gated sodium and potassium channels at densities similar to those reported experimentally for these neurons [33]–[36] had little impact on spine or shaft EPSP variability (Figure 9B; “Na^+^ and K^+^ channels"). On the other hand, adding dendritic hyperpolarization-activated cyclic-nucleotide-gated (HCN) channels [37], [38], either alone or in combination with voltage-gated sodium and potassium channels, reduced the CVs of local spine EPSP amplitude and half-width by about 38% and 35%, respectively (Figure 9B; “Na^+^, K^+^, and HCN" and “HCN only"). Dendritic HCN channels had only a small impact on the variability of shaft EPSPs, reducing the CV for EPSP amplitudes by ∼5%, and actually increasing the CV of EPSP half-widths by ∼10%. These data indicate that dendritic HCN channels, but not voltage-gated sodium and potassium channels, act synergistically with spine morphology to preferentially reduce location-dependent variability of local EPSPs occurring in dendritic spines.

Dendritic expression of HCN channels has two important and related effects on dendritic properties. HCN channels increase dendritic membrane conductance while at the same time depolarizing the dendritic membrane potential [37]–[40]. To test the relative impact of these two consequences of dendritic HCN expression on EPSP properties, we constructed two additional models: one in which the reversal potential of the HCN conductance was set to the somatic resting membrane potential (−79 mV), which eliminates HCN-mediated distance-dependent depolarization (Figure 9B; “HCN-only, E_HCN_ = −79 mV"), and another model lacking active channels, but where dendritic compartments were artificially depolarized to the same extent as occurs when HCN channels are present (Figure 9B; “Passive, HCN-like depolarization"). Setting the reversal potential for the HCN conductance to −79 mV effectively eliminated HCN-dependent reduction in spine EPSP amplitude variability, but enhanced the standardization of EPSP half-width (Figure 9B). On the other hand, depolarizing dendritic compartments in the absence of HCN mimicked the HCN-induced reduction in EPSP amplitude variability, but eliminated the influence of HCN channels on EPSP half-widths (Figure 9B). These results indicate that dendritic HCN channels reduce local spine EPSP amplitude variability via a depolarization-dependent reduction in EPSP driving force at distal locations, whereas the variability of spine EPSP half-width is reduced primarily via an HCN-mediated increase in distal dendritic membrane conductance.

### Spines enhance synapse-driven action potential generation

**Figure 10:**
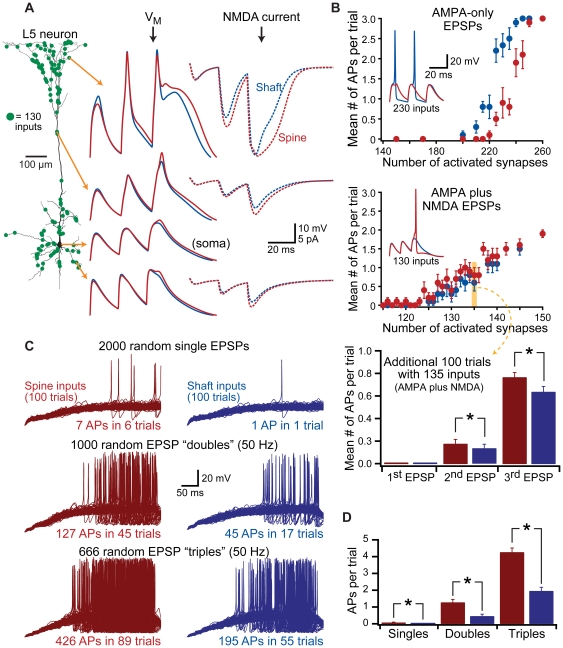
Spines enhance action potential generation. A) Left, model layer 5 neuron with spine and shaft inputs located at ∼2 µm intervals along dendrites. Green dots indicate the locations of 130 randomly selected synaptic inputs. Middle, responses to trains of 3 AMPA+NMDA EPSPs (50 Hz) delivered simultaneously to spines (red) or shafts (blue), and recorded at the indicated somatic and dendritic locations. Right, NMDA currents for the indicate synapses (dotted lines). B) Plots of the number of action potentials generated per trial vs number of coactivated synapses for AMPA-only inputs (top) and AMPA+NMDA inputs (middle). Ten trials per synapse number. Data shown as mean ± SEM. Insets show example somatic recordings from 230 AMPA-only inputs (top) or 130 AMPA+NMDA inputs (middle; different set of 130 inputs than shown in A). Lower graph shows the mean number of action potentials occurring in an additional 100 trials having 135 random AMPA+NMDA inputs. C) Superimposed responses from 100 trials in which varying numbers of synapses were activated at random timings over 400 ms at spine (left) or corresponding shaft (right) locations in the model shown in A. Each pair of trials (spine and shaft) involved the same input locations and timings. Top traces show responses to 2000 inputs activated once within the 400 ms window (random timings). Middle traces show responses to 1000 inputs activated twice each with 20 ms intervals (total of 2000 synaptic activations). Bottom traces are responses to 666 inputs activated three times with 20 ms intervals (total of 1998 synaptic activations). D) Summary graph showing the number of action potentials generated per trial for the data shown in C.

Our data demonstrate that spines enhance the activation of voltage-sensitive conductances at the synapse, promoting greater depolarization of the soma. This suggests that inputs onto spines may be more efficient than shaft inputs in generating action potentials. We tested the impact of spines on action potential generation in an active model of the layer 5 pyramidal neuron expressing Na^+^, K^+^, and HCN conductances, and having spine and shaft inputs positioned at ∼2 µm intervals along all dendrites (Figure 10A). Variable numbers of randomly selected inputs were coactivated three times at 50 Hz. For each number of synaptic inputs (e.g., 130 inputs, shown as green dots in Figure 10A), ten trials were performed, each with a different set of randomly determined input locations. Trials with identical synaptic locations were repeated for spine and shaft inputs, and the average numbers of action potentials generated per trial compared between the two input types.

When EPSPs were generated at synapses containing only AMPA-like conductances, inputs to shafts generated more action potentials than did inputs onto spines (Figure 10B, top graph), consistent with the idea that AMPA-like inputs onto spines produce slightly smaller somatic EPSPs (see Figure 2A and B). Further, due to poor temporal summation of fast-decaying AMPA receptor-mediated EPSPs, action potential generation occurred preferentially in response to the first EPSPs in the train (Figure 10B, top inset). In contrast, when synapses contained both AMPA- and NMDA-like conductances, synapses onto spines generated more action potentials per trial than did inputs onto dendritic shafts (Figure 10B, middle graph), with action potentials more likely to be generated by the last EPSP in the train (Figure 10B, middle inset). To confirm this advantage of spines, additional trials were performed with 100 randomized sets of 135 synaptic inputs (Figure 10B, bottom). Inputs onto spines generated an average of 0.95±0.06 action potentials per trial, while identical inputs delivered to the dendritic shaft resulted in only 0.78±0.07 action potentials per trial (p<0.001, paired t-test for number of spikes per trial; p<0.05, Fisher's Exact Test for spike probability).

Finally, to test the functional impact of spines during more realistic synaptic activation, stochastic patterns of synaptic input were delivered to spine or shaft inputs containing both AMPA- and NMDA-like conductances (Figure 10C and D). In the first model, 2000 randomly selected inputs were activated once at stochastic timings during a 400 ms trial. When inputs were delivered to spines, 7 action potentials were generated in 6 trials (6% of trials). When identical input locations and timings were delivered to the dendritic shaft, a single action potential was generated in only one trial (1% of trials). In a second set of simulations, 1000 synaptic inputs were activated twice, at 50 Hz, starting at random timings constrained to the first 380 ms of the 400 ms trial. Thus, the same total number of synaptic inputs were activated as in the first set of simulations (2000 inputs over 400 ms). When synapses were activated twice, action potentials were generated in 45% of trails when inputs occurred on spines, but in only 17% of trials when inputs occurred on shafts. Further, paired inputs onto spines resulted in significantly greater numbers of action potentials per trial (1.27±0.2 action potentials) compared to identical inputs onto dendritic shafts (0.45±0.1 action potentials per trial; p<0.001 when comparing spine vs shaft inputs). Finally, when 666 inputs were activated 3 times at 50 Hz (1998 total inputs over 400 ms) starting at randomly distributed timings over the initial 360 ms of the 400 ms trial, synapses on spines generated more trials with action potentials (89% of trials), and more action potentials per trial (4.26±0.3 spikes), than did inputs onto dendritic shafts (action potentials on 55% of trials and 1.95±0.2 action potentials per trial; p<0.001). These results demonstrate that spines can enhance the capacity of synaptic input to generate action potential output.

### Spine morphology and synaptic plasticity

**Figure 11:**
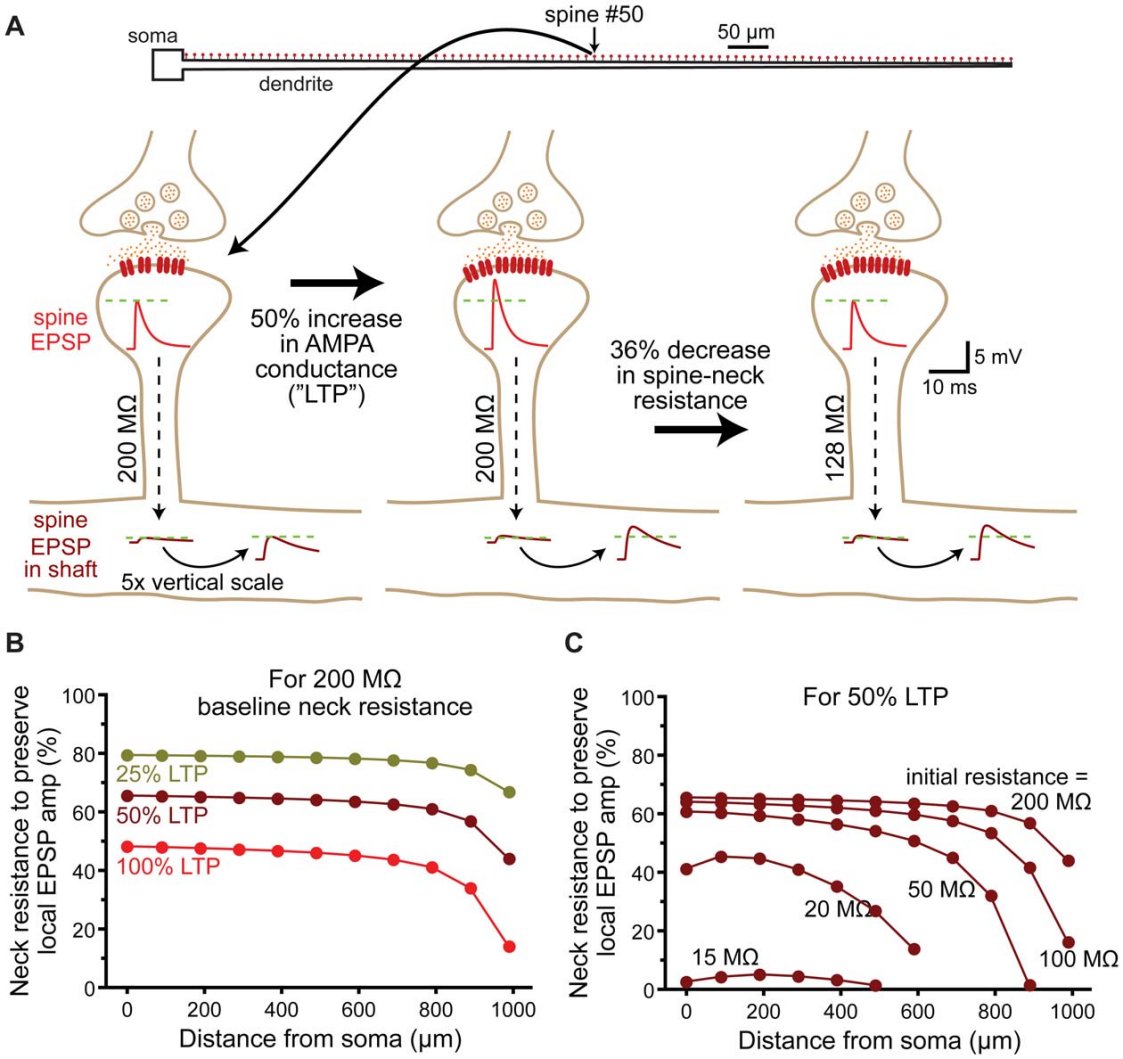
Regulation of spine neck resistance maintains local EPSP amplitude during synaptic plasticity. A) Top, ball-and-stick model neuron with 100 spines (200 MΩ neck resistance) along the dendrite. Below, a spine located half-way along the dendrite is depicted experiencing induction of long-term potentiation (LTP). Under baseline conditions, a 500 pS AMPA conductance generates a 7.7 mV EPSP in the spine head and a 0.7 mV EPSP in the dendritic shaft. Following LTP induction, the AMPA conductance is increased to 750 pS (50% LTP), increasing the local EPSP in the spine head to 11.1 mV, while the EPSP in the shaft increases to 1.04 mV. If the spine neck resistance is reduced by 36% (to 128 MΩ) the EPSP amplitude in the spine head returns to 7.7 mV, while the shaft response to the same spinous input increases marginally to 1.08 mV. B) Plots of the decrease in spine neck resistance (% of control) required to preserve local EPSP amplitude in the spine head vs dendritic location for “LTP-like" increases in AMPA conductance of 25, 50, and 100%. C) Plots of the decrease in spine neck resistance (% of control) required to preserve local EPSP amplitude following a 50% increase in AMPA conductance for spines with different initial spine neck resistances.

Spine morphology is dynamic and can rapidly change following the induction of long-term potentiation (LTP). Protocols that induce LTP increase spine volume, decrease spine neck length, and increase spine neck width [41]–[45] in an actin-dependent manner [46]. Given that spine neck resistance is a primary factor determining local EPSP amplitude in the spine head (see Figure 2D), we hypothesized that, following LTP, spines dynamically regulate their spine neck resistance to maintain a stable local EPSP amplitude in response to the potentiated synaptic conductance (Figure 11A). We tested this hypothesis by determining the extent to which spine neck resistance would need to change to maintain a constant local EPSP amplitude in the spine head following variable increases in AMPA receptor conductance, as occurs during LTP (Figure 11B and C). At most dendritic locations, only moderate decreases in spine neck resistance were required to maintain constant local EPSP amplitudes (Figure 11B; 40 to 80% of the initial value). As expected, larger increases in synaptic conductance, corresponding to greater levels of LTP, required larger reductions of spine neck resistance to maintain a uniform EPSP amplitude in the spine head. The required change in spine neck resistance was also dependent on synapse location, with more distal inputs needing greater decreases in spine neck resistance to compensate for a given increase in synaptic conductance (Figure 11B and C). This occurs because the local input impedance of the dendritic shaft increases with distance (see Figure 1D), which progressively decreases the relative contribution of spine neck resistance to the total “in series" impedance experienced by the synaptic current. Similarly, because low resistance spine necks contribute proportionally less to the total impedance, spines with low initial neck resistances require proportionally larger reductions in neck resistance to compensate for a given increase in synaptic conductance (Figure 11C). Despite these location and spine neck resistance-dependent effects, our simulations indicate that, at most dendritic locations, spines with initial neck resistances greater than 50 MΩ require only moderate changes in neck resistance to maintain a uniform local EPSP amplitude following changes in synaptic strength.

**Figure 12:**
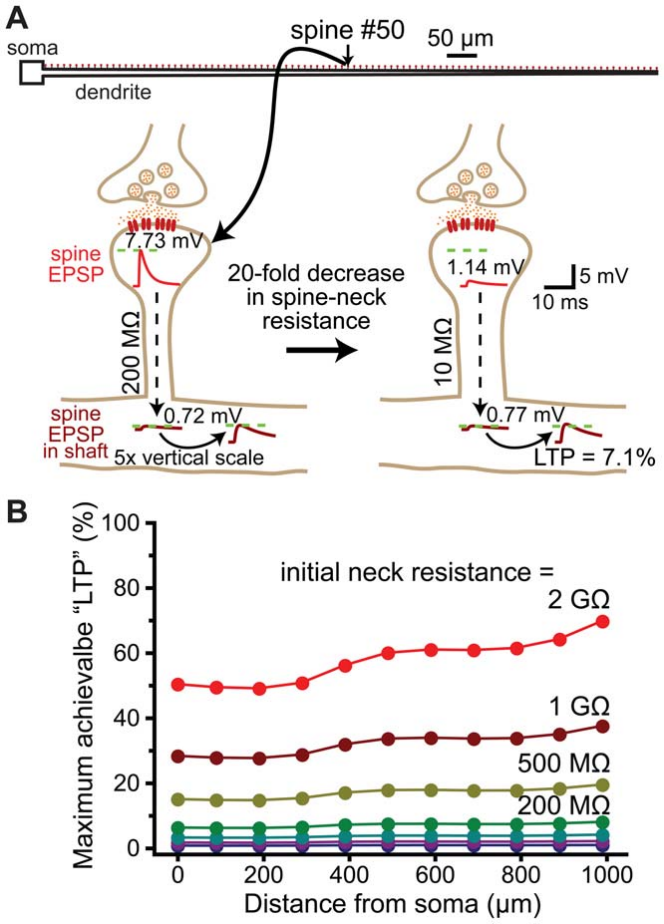
Regulation of spine neck resistance does not generate robust changes in synaptic efficacy. A) Top, ball-and-stick model neuron with 100 spines (200 MΩ neck resistance) along the dendrite. Below, a spine located half-way along the dendrite is depicted experiencing a reduction in spine neck resistance in the absence of an increase in synaptic conductance. In this case, a baseline EPSP generates a 7.73 mV EPSP at the spine head, and a 0.72 mV EPSP in the dendritic shaft. Following a 20-fold decrease in spine neck resistance, the same synaptic conductance generates a 1.14 mV EPSP at the spine head, and a 0.77 mV EPSP in the dendritic shaft: a boost of synaptic efficacy (“LTP") of 7.1%. B) Plots of the maximum possible synaptic potentiation (LTP) achievable by reducing spine neck resistance to effectively zero vs distance from the soma for a variety of initial spine neck resistances.

Can dynamic regulation of spine neck resistance influence somatic EPSP amplitude, and thereby provide a mechanism for changing synaptic efficacy, as originally proposed by Chang [1]? We tested this hypothesis by determining the maximum possible increase in somatic EPSP amplitude (% LTP) that could be generated by decreasing spine neck resistance from initial values to effectively zero (Figure 12). These drastic reductions in spine neck resistance proved relatively inefficient at boosting somatic EPSP amplitudes, with maximum increases in somatic EPSP amplitudes found to be less than 20% for spines with initial neck resistances at or below 500 MΩ (Figure 12B). These data suggest dynamic regulation of spine neck resistance during synaptic plasticity is better suited to regulation of local EPSP amplitude (see Figure 11), rather than as a mechanism for modifying synaptic efficacy.

## Discussion

Our results demonstrate that dendritic spines limit location-dependent variability of EPSP amplitude and kinetics at the site of synaptic input, and can enhance and standardize the activation of voltage-sensitive conductances at the synapse. One likely physiological consequence of this is more consistent calcium influx at the synapse, independent of location in the dendritic tree. Active conductances within dendrites enhance spine-dependent EPSP standardization and facilitate action potential generation in response to synaptic input. Given that most, if not all, of the electrical consequences of spines provide computational advantages to neurons, we propose that standardization of EPSP properties at the site of synaptic input may be a primary function of spines. Further, we propose that dynamic regulation of spine neck geometry, as observed during activity-dependent changes in synaptic strength [41]–[44], [46], allows synapses to preserve local EPSP properties even as synaptic conductance is modified.

### Mechanisms of EPSP standardization in dendritic spines

The ability of spines to standardize EPSP amplitudes and kinetics is a natural consequence of their morphology, and does not rely on specialized membrane properties or ion channel expression. The limited surface area of spines (<1 µm^2^) generates spine head compartments with very high resistance and negligible capacitance (see Figure 1). On the other hand, dendritic compartments have larger surface areas with correspondingly lower membrane resistance and larger capacitance that contribute to their having relatively low, and location-dependent, input impedance (see Figure 1D). These differences in electrical properties generate local EPSPs within spines and dendrites having different amplitudes and sensitivities to dendritic location. EPSP duration (i.e., half-width) depends in large part on local capacitance, as EPSPs are prolonged by capacitive discharge during their falling phase. Spines, with their tiny local capacitance, generate narrower EPSPs than occur in larger dendritic shafts (see Figure 2E). Variability in dendritic morphology leads to location-dependent variability in dendritic capacitance, and therefore also in dendritic EPSP kinetics. By providing a standardized local morphology at the site of synaptic input, spines greatly constrain the impact of variable dendritic geometry on local EPSPs, allowing the synaptic membrane, and associated voltage-sensitive proteins, to experience similar synaptic depolarization regardless of their location within dendritic trees. Although the addition of dendritic active conductances, such as HCN channels (see Figure 9), can further enhance spine-dependent standardization of EPSPs, EPSP standardization itself relies solely on spine morphology and the associated spine neck resistance that links synapses on spine heads with dendrites.

### Estimates of spine neck resistance

Dendritic spines have diverse morphologies [47]. While technical limitations have prevented direct measurements of spine neck resistance, previous studies have estimated spine neck resistance using a variety of experimental approaches. Electron microscopy (EM) allows for precise measurements of spine neck geometry. Assuming spine necks have aqueous interiors with cytoplasmic resistivity of 100 Ωcm, EM measurements suggest spines have neck resistances of between 1 and 400 MΩ [48]–[50]. On the other hand, estimates of spine neck resistance based largely on the rate of molecular diffusion between the spine head and dendritic shaft have suggested values ranging from the tens of MΩs [51] to over 1 GΩ [24], [52]. Although voltage imaging lacks the sensitivity to resolve small synaptic events [25], direct optical measurements of spine EPSP amplitudes in cortical pyramidal neurons suggest an upper limit of spine neck resistance of about 500 MΩ [53]. Our simulations demonstrate that spines standardize EPSPs at the site of synaptic input over the range of spine neck resistances estimated in these prior studies (Figure 2D and E). As discussed below, this previously unrecognized attribute of dendritic spines may have important consequences for the development and maintenance of synapses.

### Importance of standardized EPSP properties

Local depolarization following AMPA receptor activation is thought to play a key role in removal of the voltage-sensitive Mg^2+^ block of NMDA receptors [24]. Spine-dependent reduction in variability of local AMPA-mediated EPSPs will therefore help standardize the extent of AMPA-dependent NMDA receptor activation, independent of synapse location in the dendritic tree (Figure 6). This aspect of spine function may be critical for synapse development and maintenance [54]. Standardization of NMDA responses is also likely to be important for synaptic plasticity. Indeed, spines are commonly found on neurons exhibiting use-dependent synaptic plasticity, and have long been thought critical for induction of synaptic plasticity, in part through their capacity to compartmentalize calcium [6]–[10]. Our data demonstrate that spines may also contribute to synaptic plasticity by standardizing the activation of postsynaptic conductances localized to the synapse. This may be especially important for the induction of spike-timing dependent plasticity (STDP), as fast AMPA-receptor kinetics constrain NMDA-mediated Ca^2+^ influx to a narrow time window [55], [56]. Spines also express voltage-gated calcium channels (VGCCs) [5], [21], [23], [57]–[59], providing an additional source of calcium influx that may be important for synapse development, maintenance, and plasticity. We found that spine-dependent standardization of AMPA-mediated responses standardizes, in turn, the activation of VGCCs at the site of synaptic input. Spines may also contain calcium-activated potassium channels, which regulate NMDA receptor activation and synaptic plasticity [60], [61]. Calcium influx through VGCCs is critical for activation of calcium-activated potassium channels in spines [23], suggesting spines may indirectly standardize synaptic potassium conductances to further regulate NMDA receptor activation.

### Regulation of spine neck resistance

The ability of spines to standardize EPSP amplitude and kinetics at the site of synaptic input may be enhanced through dynamic regulation of spine neck resistance, which, together with synaptic conductance, determines the amplitude of local EPSPs in the spine head [7]. If standardization of EPSP properties at the site of synaptic input is critical for neuronal function, spine neck resistance should be negatively correlated with the magnitude of the synaptic conductance, so as to maintain a similar local EPSP amplitude in the spine head regardless of the number of AMPA receptors present at the synapse. Indeed, spine morphology is dynamic [62], [63] and correlated with synaptic efficacy: larger synaptic conductances are associated with spines with larger heads, greater AMPA receptor expression, and shorter and wider spine necks [43], [45], [64]–[67]. In the cerebral cortex and hippocampus, “mushroom" spines with larger postsynaptic densities (presumably containing more AMPA receptors) have wider spine necks than do “thin spines" with smaller postsynaptic densities [26], [47]. Since wider and shorter spine necks lead to lower spine neck resistances, these observations suggest spines may regulate neck resistance to achieve uniform local EPSP amplitudes regardless of synaptic conductance or location in the dendritic tree. Consistent with this idea, LTP induction protocols can increase spine neck diameter and reduce spine neck length [41], [44], [68]; changes that work to reduce spine neck resistance. Our data suggest dynamic reductions in spine neck resistance may compensate locally for increases in synaptic conductance (Figure 11), but are unlikely to provide a robust mechanism to enhance synaptic efficacy (Figure 12).

### Intrinsic advantages of spine morphology

The electrical properties of spines described here may provide neurons with functional advantages. By making local EPSPs less dependent on location within the dendritic tree, spines allow more uniform synaptic activation of voltage-dependent processes at the site of synaptic input. This would be difficult to achieve without dendritic spines, requiring the expression and properties of synaptic voltage-sensitive conductances to be tuned to specific dendritic environments. While there is some evidence that this may occur [69], the intrinsic morphology of spines standardizes local EPSP properties without the need for more complicated mechanisms, and has the distinct advantage that similar postsynaptic mechanisms can be expressed at all synapses, regardless of their location in the dendritic tree. This consequence of spines, which can be fine tuned by dynamic regulation of spine neck geometry, is likely to play important roles in synapse development, maintenance, and plasticity.

## Materials and Methods

Simulations were performed using NEURON 7.2 software [70]. Neuronal morphologies utilized in this study included a simple “ball-and-stick" model consisting of a cylindrical soma (40 µm length×40 µm diameter) and a tapering dendrite (1 mm long, tapering from 5 µm to 1 µm in diameter, with 1001 segments), two different layer 5 pyramidal neurons [37], [71], a hippocampal dentate granule cell [72] (source code available from entry 95960 in ModelDB; http://senselab.med.yale.edu/neurondb), a cerebellar Purkinje neuron [73], and a striatal medium-spiny neuron [74] (available from Neuromorpho.org; cell “050803c_finaltrace"). Cytoplasmic resistivity (R_i_), specific membrane capacitance (C_m_), and specific membrane resistance (R_m_) in all models were set to 100 Ωcm, 1 µF/cm^2^, and 10,000 Ωcm^2^, respectively, and resting membrane potentials were set to −79 mV, unless otherwise stated. AMPA-like EPSPs were generated by conductance changes (max conductance = 500 pS) with a reversal potential of 0 mV and exponential rise (tau = 0.2 ms) and decay (tau = 2 ms). When present, the NMDA-like conductance was modeled as in Larkum et al. [30], with NMDA conductance = 1 nS * ((e^−t/70^−e^−t/3^)/(1+0.3e^−0.08*Vm^)), with a reversal potential of +5 mV.

Because the main focus of our study was how spines influence the location dependence of EPSPs at the site of synaptic input, it was necessary to have much finer control of spine location than is possible with spatial discretization strategies such as the d_lambda rule [75]. Therefore, we calculated the value for the discretization parameter “nseg" as the length (L) of each dendritic section (in µm) rounded up to the next larger odd integer. This produced compartments whose lengths were generally slightly less than 1 µm, and allowed placement of spines at specific locations with better than 0.5 µm precision. In the somatosensory layer 5 pyramidal neuron with active properties (Figures 9 and 10), and in the ball-and-stick neuron used to test NMDA-spike thresholds (Figure 7), spines were placed every 2 µm by attaching spines to every other compartment along a path; in the text and figures this is described as “∼2 µm intervals." In all other simulations, spines were attached to compartments that were closest to whole multiples of 10 µm from the soma. For display purposes, figures show EPSPs located at select intervals (e.g., from every 2nd, 5th, or 10th spine, corresponding to intervals of ∼20, ∼50, or ∼100 um, respectively). In all graphs in which the abscissa is distance from the soma, the values plotted are actual anatomical distances from the soma, as calculated by the distance() function in NEURON. Z_N_ for the ball-and-stick model (Figure 1D) was calculated using NEURON's impedance class functions with an input frequency of 100 Hz.

Excitatory synaptic conductances were positioned on spine heads or on dendritic shafts opposite spines. Spine necks were 1 µm long and spine heads had diameters and lengths of 500 nm. Spine neck resistance was adjusted either by modifying spine neck diameter (Figure 2), or by changing the cytoplasmic resistivity of spine necks having diameters of 80 nm (all other simulations). Similar results were obtained with both methods. In some models, voltage-sensitive calcium conductances were localized to small compartments (0.01 um tall, 0.25 µm diameter) placed on spine heads or shafts. In active models (Figures 9 and 10), fast-inactivating voltage-gated sodium channels and delayed-rectifier potassium channels [76] (source code available from entry 2488 in ModelDB), as well as HCN channels [38], were included as indicated in the text. The density of sodium and potassium channels were set to 4,000 pS/µm^2^ and 1000 pS/µm^2^, respectively, in the axon initial segment and nodes of Ranvier [77], and to 100 pS/µm^2^ in the soma. Sodium, potassium, and HCN channels were incorporated into dendritic segments and spines in a distance dependent manner, with sodium and potassium channel densities decreasing linearly with distance from the soma [36], while HCN channel density increased exponentially with distance based on the function: y_0_+A * e^(d/λ)^, where y_0_ = −2 pS/µm^2^, A = 4.29 pS/µm^2^, λ = 324 µm, and d = distance from soma (in µm) [38]. Unless otherwise stated, the reversal potential of HCN channels (E_HCN_) was set to −45 mV. Simulations assumed a nominal temperature of 35°C.

Data are presented as mean ± SEM unless otherwise stated. Significant differences between spine and shaft EPSP measurements (defined as p<0.05) were determined using Student's t-tests for paired samples. Comparison of CVs for EPSP properties across models utilized a one-way ANOVA with Tukey-Kramer post-tests.

## References

[1] Chang HT (1952). Cortical neurons with particular reference to the apical dendrites.. Cold Spring Harbor Symposia on Quantitative Biology.

[2] Diamond J, Gray EG, Yasargil GM, Andersen P, Jensen JKS (1970). The function of the dendritic spine: an hypothesis.. Excitatory Synaptic Mechanisms.

[3] Rall W, Woody CD, Brown KA, Crow TJ, Knispel JD (1974). Dendritic spines, synaptic potency, and neuronal plasticity.. Cellular Mechanisms Subserving Changes in Neuronal Activity.

[4] Araya R, Jiang J, Eisenthal KB, Yuste R (2006). The spine neck filters membrane potentials.. Proc Natl Acad Sci U S A.

[5] Bloodgood BL, Giessel AJ, Sabatini BL (2009). Biphasic synaptic Ca influx arising from compartmentalized electrical signals in dendritic spines.. PLoS Biol.

[6] Muller W, Connor JA (1991). Dendritic spines as individual neuronal compartments for synaptic Ca2+ responses.. Nature.

[7] Koch C, Zador A (1993). The function of dendritic spines: devices subserving biochemical rather than electrical compartmentalization.. J Neurosci.

[8] Yuste R, Denk W (1995). Dendritic spines as basic functional units of neuronal integration.. Nature.

[9] Sabatini BL, Oertner TG, Svoboda K (2002). The life cycle of Ca(2+) ions in dendritic spines.. Neuron.

[10] Korkotian E, Segal M (2006). Spatially confined diffusion of calcium in dendrites of hippocampal neurons revealed by flash photolysis of caged calcium.. Cell Calcium.

[11] Ramon y Cajal S (1899). Regias y consejos sobre investigación biológica.

[12] Chklovskii DB (2004). Synaptic connectivity and neuronal morphology: two sides of the same coin.. Neuron.

[13] Segev I, London M (2000). Untangling dendrites with quantitative models.. Science.

[14] Gulledge AT, Kampa BM, Stuart GJ (2005). Synaptic integration in dendritic trees.. J Neurobiol.

[15] Rinzel J, Rall W (1974). Transient response in a dendritic neuron model for current injected at one branch.. Biophys J.

[16] Kawato M, Tsukahara N (1984). Electrical properties of dendritic spines with bulbous end terminals.. Biophys J.

[17] Wilson CJ (1984). Passive cable properties of dendritic spines and spiny neurons.. J Neurosci.

[18] Jaslove SW (1992). The integrative properties of spiny distal dendrites.. Neuroscience.

[19] Segev I, Friedman A, White EL, Gutnick MJ (1995). Electrical consequences of spine dimensions in a model of a cortical spiny stellate cell completely reconstructed from serial thin sections.. J Comput Neurosci.

[20] Kawato M, Tsukahara N (1983). Theoretical study on electrical properties of dendritic spines.. J Theor Biol.

[21] Isope P, Murphy TH (2005). Low threshold calcium currents in rat cerebellar Purkinje cell dendritic spines are mediated by T-type calcium channels.. J Physiol.

[22] Araya R, Nikolenko V, Eisenthal KB, Yuste R (2007). Sodium channels amplify spine potentials.. Proc Natl Acad Sci U S A.

[23] Bloodgood BL, Sabatini BL (2007). Nonlinear regulation of unitary synaptic signals by CaV(2.3) voltage-sensitive calcium channels located in dendritic spines.. Neuron.

[24] Grunditz A, Holbro N, Tian L, Zuo Y, Oertner TG (2008). Spine neck plasticity controls postsynaptic calcium signals through electrical compartmentalization.. J Neurosci.

[25] Peterka DS, Takahashi H, Yuste R (2011). Imaging voltage in neurons.. Neuron.

[26] Harris KM, Jensen FE, Tsao B (1992). Three-dimensional structure of dendritic spines and synapses in rat hippocampus (CA1) at postnatal day 15 and adult ages: implications for the maturation of synaptic physiology and long-term potentiation.. J Neurosci.

[27] Lester RA, Clements JD, Westbrook GL, Jahr CE (1990). Channel kinetics determine the time course of NMDA receptor-mediated synaptic currents.. Nature.

[28] Schiller J, Major G, Koester HJ, Schiller Y (2000). NMDA spikes in basal dendrites of cortical pyramidal neurons.. Nature.

[29] Nevian T, Larkum ME, Polsky A, Schiller J (2007). Properties of basal dendrites of layer 5 pyramidal neurons: a direct patch-clamp recording study.. Nat Neurosci.

[30] Larkum ME, Nevian T, Sandler M, Polsky A, Schiller J (2009). Synaptic integration in tuft dendrites of layer 5 pyramidal neurons: a new unifying principle.. Science.

[31] Branco T, Hausser M (2011). Synaptic integration gradients in single cortical pyramidal cell dendrites.. Neuron.

[32] Spruston N (2008). Pyramidal neurons: dendritic structure and synaptic integration.. Nat Rev Neurosci.

[33] Stuart GJ, Sakmann B (1994). Active propagation of somatic action potentials into neocortical pyramidal cell dendrites.. Nature.

[34] Bekkers JM (2000). Distribution and activation of voltage-gated potassium channels in cell-attached and outside-out patches from large layer 5 cortical pyramidal neurons of the rat.. J Physiol.

[35] Korngreen A, Sakmann B (2000). Voltage-gated K+ channels in layer 5 neocortical pyramidal neurones from young rats: subtypes and gradients.. J Physiol.

[36] Lorincz A, Nusser Z (2010). Molecular identity of dendritic voltage-gated sodium channels.. Science.

[37] Stuart G, Spruston N (1998). Determinants of voltage attenuation in neocortical pyramidal neuron dendrites.. J Neurosci.

[38] Kole MH, Hallermann S, Stuart GJ (2006). Single Ih channels in pyramidal neuron dendrites: properties, distribution, and impact on action potential output.. J Neurosci.

[39] Magee JC (1998). Dendritic hyperpolarization-activated currents modify the integrative properties of hippocampal CA1 pyramidal neurons.. J Neurosci.

[40] Berger T, Larkum ME, Luscher HR (2001). High I(h) channel density in the distal apical dendrite of layer V pyramidal cells increases bidirectional attenuation of EPSPs.. J Neurophysiol.

[41] Fifkova E, Anderson CL (1981). Stimulation-induced changes in dimensions of stalks of dendritic spines in the dentate molecular layer.. Exp Neurol.

[42] Lang C, Barco A, Zablow L, Kandel ER, Siegelbaum SA (2004). Transient expansion of synaptically connected dendritic spines upon induction of hippocampal long-term potentiation.. Proc Natl Acad Sci U S A.

[43] Matsuzaki M, Honkura N, Ellis-Davies GC, Kasai H (2004). Structural basis of long-term potentiation in single dendritic spines.. Nature.

[44] Kopec CD, Li B, Wei W, Boehm J, Malinow R (2006). Glutamate receptor exocytosis and spine enlargement during chemically induced long-term potentiation.. J Neurosci.

[45] Tanaka J, Horiike Y, Matsuzaki M, Miyazaki T, Ellis-Davies GC (2008). Protein synthesis and neurotrophin-dependent structural plasticity of single dendritic spines.. Science.

[46] Okamoto K, Nagai T, Miyawaki A, Hayashi Y (2004). Rapid and persistent modulation of actin dynamics regulates postsynaptic reorganization underlying bidirectional plasticity.. Nat Neurosci.

[47] Peters A, Kaiserman-Abramof IR (1970). The small pyramidal neuron of the rat cerebral cortex. The perikaryon, dendrites and spines.. Am J Anat.

[48] Harris KM, Stevens JK (1989). Dendritic spines of CA 1 pyramidal cells in the rat hippocampus: serial electron microscopy with reference to their biophysical characteristics.. J Neurosci.

[49] Nicholson DA, Trana R, Katz Y, Kath WL, Spruston N (2006). Distance-dependent differences in synapse number and AMPA receptor expression in hippocampal CA1 pyramidal neurons.. Neuron.

[50] Arellano JI, Benavides-Piccione R, Defelipe J, Yuste R (2007). Ultrastructure of dendritic spines: correlation between synaptic and spine morphologies.. Front Neurosci.

[51] Svoboda K, Tank DW, Denk W (1996). Direct measurement of coupling between dendritic spines and shafts.. Science.

[52] Bloodgood BL, Sabatini BL (2005). Neuronal activity regulates diffusion across the neck of dendritic spines.. Science.

[53] Palmer LM, Stuart GJ (2009). Membrane potential changes in dendritic spines during action potentials and synaptic input.. J Neurosci.

[54] Turrigiano GG, Nelson SB (2004). Homeostatic plasticity in the developing nervous system.. Nat Rev Neurosci.

[55] Oertner TG (2009). How do synapses measure milliseconds?. Front Comput Neurosci.

[56] Holbro N, Grunditz A, Wiegert JS, Oertner TG (2010). AMPA receptors gate spine Ca(2+) transients and spike-timing-dependent potentiation.. Proc Natl Acad Sci U S A.

[57] Sabatini BL, Svoboda K (2000). Analysis of calcium channels in single spines using optical fluctuation analysis.. Nature.

[58] Lee SJ, Escobedo-Lozoya Y, Szatmari EM, Yasuda R (2009). Activation of CaMKII in single dendritic spines during long-term potentiation.. Nature.

[59] Leitch B, Szostek A, Lin R, Shevtsova O (2009). Subcellular distribution of L-type calcium channel subtypes in rat hippocampal neurons.. Neuroscience.

[60] Faber ES, Delaney AJ, Sah P (2005). SK channels regulate excitatory synaptic transmission and plasticity in the lateral amygdala.. Nat Neurosci.

[61] Ngo-Anh TJ, Bloodgood BL, Lin M, Sabatini BL, Maylie J (2005). SK channels and NMDA receptors form a Ca2+-mediated feedback loop in dendritic spines.. Nat Neurosci.

[62] Fischer M, Kaech S, Knutti D, Matus A (1998). Rapid actin-based plasticity in dendritic spines.. Neuron.

[63] Dunaevsky A, Tashiro A, Majewska A, Mason C, Yuste R (1999). Developmental regulation of spine motility in the mammalian central nervous system.. Proc Natl Acad Sci U S A.

[64] Matsuzaki M, Ellis-Davies GC, Nemoto T, Miyashita Y, Iino M (2001). Dendritic spine geometry is critical for AMPA receptor expression in hippocampal CA1 pyramidal neurons.. Nat Neurosci.

[65] Kasai H, Matsuzaki M, Noguchi J, Yasumatsu N, Nakahara H (2003). Structure-stability-function relationships of dendritic spines.. Trends Neurosci.

[66] Lamprecht R, LeDoux J (2004). Structural plasticity and memory.. Nat Rev Neurosci.

[67] Korkotian E, Segal M (2007). Morphological constraints on calcium dependent glutamate receptor trafficking into individual dendritic spine.. Cell Calcium.

[68] Otmakhov N, Tao-Cheng JH, Carpenter S, Asrican B, Dosemeci A (2004). Persistent accumulation of calcium/calmodulin-dependent protein kinase II in dendritic spines after induction of NMDA receptor-dependent chemical long-term potentiation.. J Neurosci.

[69] Katz Y, Menon V, Nicholson DA, Geinisman Y, Kath WL (2009). Synapse distribution suggests a two-stage model of dendritic integration in CA1 pyramidal neurons.. Neuron.

[70] Carnevale NT, Hines ML (2006). The NEURON Book.

[71] Jaffe DB, Carnevale NT (1999). Passive normalization of synaptic integration influenced by dendritic architecture.. J Neurophysiol.

[72] Schmidt-Hieber C, Jonas P, Bischofberger J (2007). Subthreshold dendritic signal processing and coincidence detection in dentate gyrus granule cells.. J Neurosci.

[73] Roth A, Hausser M (2001). Compartmental models of rat cerebellar Purkinje cells based on simultaneous somatic and dendritic patch-clamp recordings.. J Physiol.

[74] Martone ME, Gupta A, Wong M, Qian X, Sosinsky G (2002). A cell-centered database for electron tomographic data.. J Struct Biol.

[75] Hines ML, Carnevale NT (2001). NEURON: a tool for neuroscientists.. Neuroscientist.

[76] Mainen ZF, Sejnowski TJ (1996). Influence of dendritic structure on firing pattern in model neocortical neurons.. Nature.

[77] Kole MH, Ilschner SU, Kampa BM, Williams SR, Ruben PC (2008). Action potential generation requires a high sodium channel density in the axon initial segment.. Nat Neurosci.

